# Frailty diminishes functional outcome in patients with nonaneurysmal subarachnoid hemorrhage: a dual specialized neurovascular center analysis

**DOI:** 10.1007/s00415-025-13227-5

**Published:** 2025-07-02

**Authors:** Larissa Penner, Anna-Laura Potthoff, Tim Lampmann, Rebecca Heinz, Johannes Lemcke, Motaz Hamed, Florian Gessler, Hartmut Vatter, Patrick Schuss, Alexis Hadjiathanasiou, Matthias Schneider

**Affiliations:** 1 Department of Neurosurgery, BG Klinikum Unfallkrankenhaus Berlin gGmbH, Bonn, Germany; 2 Department of Neurosurgery, University Hospital Bonn, Venusberg Campus 1, 53127 Bonn, Germany; 3 Department of Neurosurgery, University Medicine Rostock, Rostock, Germany

**Keywords:** Nonaneurysmal SAH, Subarachnoid hemorrhage, Frailty

## Abstract

**Objective:**

Frailty is increasingly recognized as a significant prognostic factor in various conditions. However, its impact on outcomes following spontaneous, nonaneurysmal subarachnoid hemorrhage (naSAH) remains unclear. This study aimed to assess the association between pre-existing frailty and functional outcomes in patients with naSAH.

**Methods:**

The study cohort was made up of 257 patients treated for naSAH at two neurovascular centers between 2012 and 2021. Frailty prior to naSAH was assessed using the modified frailty index (mFI), with patients classified as nonfrail (mFI 0–1) or frail (mFI ≥ 2). Functional outcomes at 6 months were evaluated using the modified Rankin Scale (mRS), categorized as favorable (mRS 0–2) or unfavorable (mRS 3–6). A multivariable logistic regression analysis was performed to identify independent predictors of unfavorable outcomes.

**Results:**

Among 257 naSAH patients, 56 (22%) were classified as frail (mFI ≥ 2) before ictus. At the 6-month follow-up, unfavorable outcomes were observed in 17 of the 56 frail patients (30%) compared to 21 of 201 nonfrail patients (10%) (*p* = 0.001). In addition to established negative prognostic factors such as delayed cerebral ischemia (*p* < 0.001) and poor-grade naSAH (Hunt & Hess grades III–IV; *p* = 0.001), multivariable analysis identified frailty (*p* = 0.03) as an independent and significant predictor of unfavorable functional outcomes.

**Conclusions:**

Frailty prior to hemorrhage, as determined by an mFI of ≥ 2, was associated with poor functional outcomes at 6 months in patients with naSAH. These findings underscore the importance of incorporating frailty assessments into early prognostic evaluations to guide patient management and counseling.

## Introduction

Up to 20% of patients with neuroimaging evidence of basal subarachnoid hemorrhage (SAH) exhibit a normal cerebral angiogram during further clarification of the bleeding source [1]. These patients, classified as having spontaneous nonaneurysmal SAH (naSAH), generally have a more favorable prognosis as compared to those with spontaneous aneurysmal SAH (aSAH) [2]. This improved outcome is attributed to a lower incidence of symptomatic cerebral vasospasm (CVS) and a reduced risk of secondary brain injury associated with delayed cerebral ischemia (DCI) in naSAH cases [3].

Nevertheless, poor functional outcome remains an issue in naSAH [4]. Different efforts to identify (early) predictive factors for poor outcome in naSAH have been pursued in the past [5–7]. Konzcalla et al. most recently reported that naSAH continues to be a rare disease overall, but nevertheless noted an increase in naSAH incidence over the past 15 years [8]. Among the identified prognostic factors, patient age has been shown to be a critical determinant of poor functional outcomes.

In the recent years, frailty has emerged as an age-independent and more precise variable in various prognostic assessments [9–12]. To the best of the authors’ knowledge, studies on the influence of frailty on neurological/functional outcome after naSAH are currently not available. This study aimed to investigate the potential prognostic value of frailty in patients with spontaneous, nonaneurysmal SAH by analyzing data from two specialized neurosurgical centers.

## Methods

### Patient population

We retrospectively included 257 consecutive adult patients from two specialized neurovascular centers (BG Klinikum Unfallkrankenhaus Berlin and University Hospital Bonn, Germany) who suffered from spontaneous naSAH between 2012 and 2021. Computed tomography (CT) and/or lumbar puncture was obtained to determine the diagnosis of SAH in principle. Patients with spontaneous SAH were divided into two groups based on their clinical condition on admission: Patients with good grade SAH (Hunt and Hess (HH) grades I–II) versus (vs.) patients with poor grade SAH (HH grades III–V). All patients with spontaneous SAH underwent four-vessel digital subtraction angiography (DSA) to exclude aneurysmal or other sources of hemorrhage. Those patients with a detected origin of hemorrhage (e.g., intracranial aneurysm, vascular malformation) or traumatic cause of SAH were excluded from further analysis. For angiogram-negative SAH cases, additional spinal magnetic resonance imaging (MRI) was conducted to exclude any spinal bleeding source. In addition, a second four-vessel DSA was performed within 6 weeks of ictus according to the respective guidelines of the participating neurovascular centers. Patients with spontaneous, nontraumatic SAH without identification of a bleeding source after the above-mentioned diagnostic work-up were then included in the present study for further analysis. Patients with isolated convexity SAH were not included in this study.

Cerebral vasospasm (CVS) was monitored and designated as symptomatic CVS in the presence of delayed ischemic neurologic deficit (DIND) or vasospasm-related perfusion deficits on CT perfusion scans performed. Symptomatic CVS were treated by induced hypertension [13]. Delayed cerebral ischemia (DCI) was defined as the appearance of new ischemic lesions on radiologic imaging during the course of treatment. Shunt-dependent hydrocephalus was identified in patients who required permanent cerebrospinal fluid (CSF) drainage through a ventriculoperitoneal shunt (VPS) due to persistent hydrocephalus despite attempts of weaning.

Functional outcomes were assessed using the modified Rankin Scale (mRS) 6 months after naSAH and divided into favorable (mRS 0–2) and unfavorable (mRS 3–6) categories.

### Modified frailty index (mFI)

The modified Frailty Index (mFI-5) was used to assess frailty prior to naSAH in patients. The mFI-5 is a shortened version of the mFI-11 and consists of five nonoverlapping clinical factors: diabetes mellitus, functional status (not independent), chronic obstructive pulmonary disease or pneumonia, congestive heart failure, and medically treated hypertension [14]. Each factor is assigned a score of 1 if present, resulting in a minimum score of 0 and a maximum score of 5 [14]. For analysis purposes, patients were categorized into two groups: non/moderately frail (mFI-5 0–2) and frail (mFI-5 ≥ 2) [15].

### Statistical analysis

Statistical data analysis was conducted using the SPSS software package (SPSS, version 27, IBM Corp., Armonk, NY, USA). Associations between parametric variables were analyzed using unpaired, two-tailed Student’s *t* test. The Mann–Whitney *U* test was employed for comparison of continuous variables. Categorical variables were compared using Chi-square test or Fisher’s exact test. Statistical significance was defined as *p* < 0.05. To further elucidate the influence of frailty on naSAH patient outcomes, a multivariable regression analysis was performed, incorporating relevant variables to determine the independent effect of frailty on the prognosis in nonaneurysmal SAH patients.

## Results

### Patient and disease-related characteristics

The study cohort included 257 patients with spontaneous naSAH. At the time of ictus, the median age was 57 years (interquartile range (IQR) 49–65), with 117 patients (46%) being female. Upon presentation, the median HH grade was 1 (IQR 1–2), and the median Fisher grade was 3 (IQR 2–3). Delayed cerebral ischemia (DCI) occurred in 14 patients (5%) during hospitalization. At the 6-month follow-up, 42 patients (16%) exhibited unfavorable outcomes, in terms of a mRS score of 3–6. The median mFI at admission was 1 (IQR 0–1). For additional details, refer to Table 1.

**Table 1:** Patient characteristics*

	*n* = 257
Median age (IQR)	57 (49–65)
Female sex	117 (4)
Median HH grade (IQR)	1 (1–2)
Median Fisher grade (IQR)	3 (3–3)
Mere prepontine SAH	60 (23%)
Occurrence of DCI	14 (5%)
Shunt-dependent hydrocephalus	28 (11%)
Median mFI (IQR)	1 (0–1)
Unfavorable outcome (mRS 3–5)	42 (16%)

*Values represent number of patients unless otherwise indicated (%)

*DCI* delayed cerebral ischemia, *HH* Hunt and Hess classification, *IQR* interquartile range, *mFI* modified frailty index, *mRS* modified Rankin Scale, *SAH* subarachnoid hemorrhage

### Influence of frailty on functional outcome following naSAH

Among the 257 naSAH patients analyzed, 56 (21%) were classified as frail, while 201 (79%) were categorized as nonfrail (Table 2). In this subgroup, the following components of the frailty index were present: arterial hypertension (*n* = 53, 95%), impaired functional independence (*n* = 37, 66%), diabetes mellitus (*n* = 28, 50%), congestive heart failure (*n* = 13, 23%), and chronic obstructive pulmonary disease (*n* = 7, 13%). Within the frail group, 34 of 56 patients (61%) were aged ≥ 65 years, compared to 35 of 201 patients (17%) in the nonfrail group (*p* < 0.001). At admission, 12 frail patients (21%) presented with HH grades III–V, as compared to 18 nonfrail patients (9%) (*p* = 0.02). The presence of mere prepontine SAH and the occurrence of DCI did not differ significantly differ between frail and nonfrail patients (*p* = 0.16 and *p* = 0.51, respectively).

**Table 2:** Patients with naSAH stratified for frailty*

	mFI < 2 *n* = 201	mFI ≥ 2 *n* = 56	*p* value
Patient age > = 65 yrs	35 (17)	34 (61)	< 0.001
Female sex	96 (47)	22 (39)	0.36
HH grade III–V	18 (9)	12 (21)	0.02
Mere prepontine SAH	51 (25)	9 (16)	0.16
Occurrence of DCI	10 (5)	4 (7)	0.51
Shunt-dependent hydrocephalus	18 (9)	10 (18)	0.09
Unfavorable outcome (mRS 3–5)	20 (10)	22 (39)	< 0.0001

*Values represent number of patients unless otherwise indicated (%)

*DCI* delayed cranial ischemia, *HH* Hunt and Hess classification, *mFI* modified frailty index, *mRS* modified Rankin Scale, *naSAH* nonaneurysmal subarachnoid hemorrhage, *OR* odds ratio, *yrs* years

**Fig. 1:**
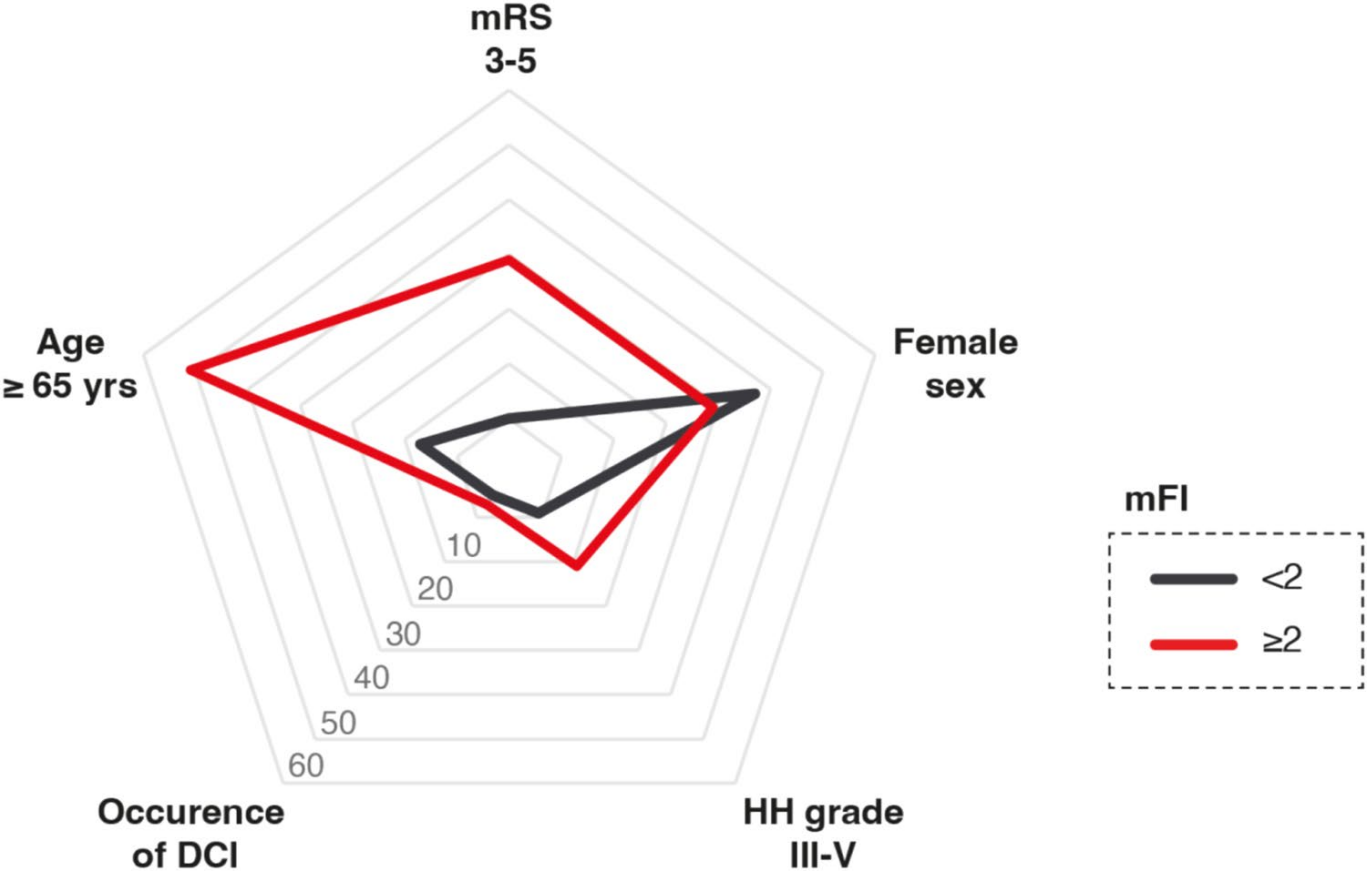
Radar plot depicts patient- and disease-related characteristics dependent on mFI < 2 and mFI ≥ 2 for patients with naSAH. *mFI* modified frailty index, *naSAH* nonaneurysmal subarachnoid hemorrhage

**Fig. 2:**
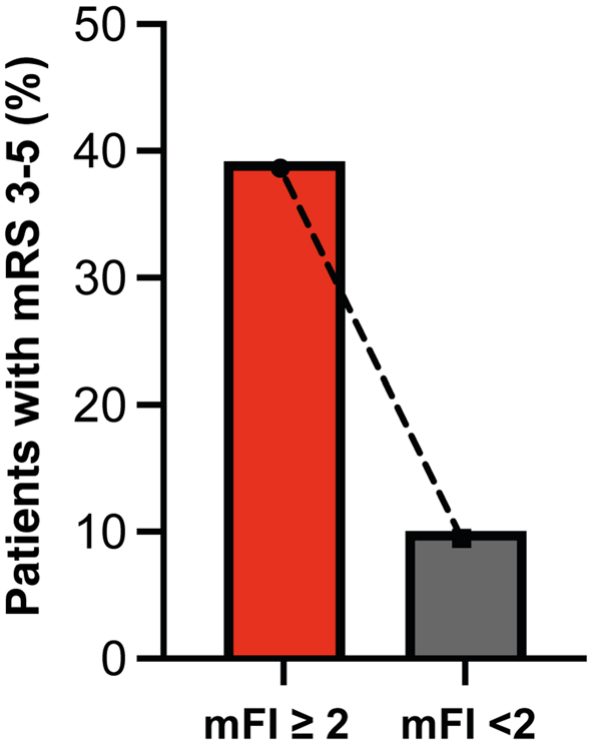
Bar plots depict functional outcome dependent on mFI < 2 and mFI ≥ 2 for patients with naSAH. *mFI* modified frailty index, *naSAH* nonaneurysmal subarachnoid hemorrhage

Frail patients were significantly more likely to experience unfavorable outcomes at 6 months, objectified by a mRS score of 3–5: unfavorable outcomes were observed in 22 of 56 frail patients (39%) vs. 20 of 201 nonfrail patients (10%) (*p* < 0.0001, OR 5.9, 95% CI 2.9–11.9) (Table 2; Figs. 1 and 2).

Among patients with mFI ≥ 2, 7 of 56 (13%) had a premorbid mRS of 3–5. In contrast, 3 of 201 (2%) patients with mFI < 2 had a premorbid mRS of 3–5 (*p* < 0.001).

### Multivariable analysis identifies frailty as a risk factor for unfavorable outcome in naSAH

A multivariable logistic regression analysis was performed to identify significant and independent predictors of unfavorable outcome at the 6 months follow-up in patients with naSAH. The multivariable logistic regression model included the following covariates: age < 65 years, female sex, Hunt and Hess grade 3–5, mere prepontine SAH, occurrence of DCI, and mFI ≥ 2. The analysis revealed that *“*HH grade III–V*”* (*p* = 0.001, OR 20.5, 95% CI 3.4–125.0), *“*occurrence of DCI*”* (*p* < 0.001, OR 96.2, 95% CI 7.8–1179), and *“*frailty (mFI ≥ 2)*”* (*p* = 0.03, OR 7.6, 95% CI 1.2–46.3) were each independently associated with an increased likelihood of unfavorable functional outcomes. The model accounted for 57% of the variance in outcomes (Nagelkerke’s R^2^ = 0.57, Table 3).

**Table 3:** Multivariable analysis identifies mFI ≥ 2 as a risk factor for unfavorable outcome in naSAH

	Adjusted OR	95% CI	*p* value
Age < 65 years	0.7	0.1–5.0	0.7
Female sex	0.6	0.1–3.3	0.6
HH grade III–V	20.5	3.4–125.0	**0.001**
Mere prepontine SAH	2.1	0.1–34.7	0.6
Occurrence of DCI	96.2	7.8–1179.0	** < 0.001**
*mFI* ≥ *2*	*7.6*	*1.2–46.3*	***0.03***

*CI* confidence interval, *DCI* delayed cranial ischemia, *IQR* interqartile range, *mFI* modified frailty index, *naSAH* nonaneurysmal subarachnoid hemorrhage, *OR* Odds ratio

To account for the limited number of outcome events and to reduce the risk of model overfitting, a simplified multivariable logistic regression analysis was performed including only three covariates: HH grade, occurrence of DCI, and frailty. In this reduced model, ‘HH grade III-V’ (*p* < 0.001, OR 18.7, 95% CI 7.0–49.7), ‘occurrence of DCI’ (*p* < 0.001, OR 34.5, 95% CI 8.6–138.8), and ‘frailty (mFI ≥ 2)’ (*p* = 0.005, OR 3.8, 95% CI 1.5–9.6) were each found to be significantly associated with unfavorable functional outcome. The model explained 44% of the variance in outcomes (Nagelkerke’s R^2^ = 0.44).

## Discussion

The present study investigates the impact of frailty on functional outcomes in patients with naSAH. Our findings suggest that frailty prior to hemorrhage, as measured by the mFI, is associated with an increased likelihood of poor functional outcomes, even when adjusted for other relevant clinical factors. Specifically, frail patients (mFI ≥ 2) exhibited a markedly higher rate of unfavorable outcomes at the 6-month follow-up as compared to nonfrail patients (mFI < 2). These results highlight the critical need to incorporate frailty assessments into the early prognostic evaluation of naSAH patients.

Frailty, as quantified by the mFI, has been increasingly recognized as a critical determinant of outcomes in various medical conditions, including cardiovascular diseases and cancer [16–18]. Notably, it has also been shown that frailty plays a significant role in the outcomes of patients with aneurysmal subarachnoid hemorrhage (SAH) [9]. Our study is the first to specifically examine the role of frailty in naSAH outcomes even when accounting for other known prognostic factors such as age [8], Hunt and Hess grade [19], and the occurrence of DCI [20].

The relationship between frailty and poor outcomes in the field of SAH may be attributed to several factors. Frail patients often have a diminished physiological reserve [10, 21, 22], which may hinder their ability to recover from the acute stress of a hemorrhagic event. In addition, frailty is associated with a higher prevalence of comorbid conditions [23], which can complicate the clinical course and management of naSAH. Previous research on prognostication in naSAH has largely focused on traditional predictors such as age [8], clinical grading scales [2], and radiological findings [3]. However, frailty may provide a more nuanced and comprehensive approach to risk stratification. Unlike age, which is a single-factor predictor [24], frailty encompasses multiple dimensions of health, including comorbidities, functional status, and overall physiological resilience [25]. In this context, studies in other fields have shown that frailty is a stronger predictor of adverse outcomes than age alone, as it captures the cumulative burden of health deficits that may not be fully reflected by age or other single metrics [26–28]. Interestingly, 39% of frail patients in our cohort were under the age of 65. This finding highlights that frailty is not synonymous with advanced chronological age but rather reflects a multidimensional vulnerability affecting patients across age groups. As such, frailty screening using tools like the mFI may add prognostic value even in younger patients, where clinical risk is often underestimated based on age alone. This supports a broader application of frailty assessment in the early evaluation of naSAH patients, regardless of age. The mFI, while primarily based on comorbidities and functional status, has been validated across surgical and neurosurgical populations and is well suited for retrospective analysis. We acknowledge that instruments such as the Clinical Frailty Scale (CFS) may provide a broader assessment of frailty and could complement or refine risk stratification in prospective studies. Furthermore, although premorbid mRS was not formally included in the multivariable model, the number of functionally dependent patients prior to hemorrhage was low, and this potential confounder has been discussed accordingly. Importantly, age is not part of the mFI and was analyzed separately to avoid collinearity. It is important to clarify that the concepts of functional independence in the mFI and functional outcome as assessed by the mRS are not directly equivalent. Although the mFI includes a component related to independence in activities of daily living—which may overlap with the concept of functional outcome assessed via the mRS—only 12.5% of frail patients in our cohort had a premorbid mRS of 3 or higher. This suggests that frailty, as measured by the mFI, was not solely driven by baseline functional status but also reflected other relevant health deficits. Therefore, the mFI provides additional prognostic information beyond the premorbid mRS alone. This more holistic approach may be particularly valuable in neurovascular conditions like SAH, where the interplay of multiple health factors are known to significantly influence recovery and long-term outcomes [29]. Furthermore, given that long-term outcomes are generally more favorable in naSAH compared to aneurysmal SAH, frailty may be particularly valuable as an additional tool for risk stratification in naSAH patients [30, 31]. In this context, the degree of frailty may offer a beneficial additional layer of prognostication in naSAH, helping to refine risk stratification and ultimately guide patient management more effectively.

Given the increasing prevalence of naSAH in older populations [29], who are more likely to be frail, our findings underscore the need for clinicians to incorporate frailty assessments into routine clinical practice. The use of the mFI in routine evaluations may aid in identifying high-risk patients who could benefit from more intensive monitoring and tailored therapeutic interventions. In general surgery, the frailty index has already been recognized as an important predictive variable, particularly in emergency procedures for patients over 60 years of age [32]. The modified frailty index can effectively evaluate the risk of both morbidity and mortality in these patients, serving as a valuable preoperative risk assessment tool for acute care surgeons [32]. Extending the application of the mFI to neurovascular care could similarly enhance the accuracy of prognostic models and guide treatment decisions for naSAH patients. As such, frail patients might require more aggressive management of complications such as DCI or hydrocephalus, as well as closer follow-up after discharge to optimize outcomes in the long-term. Further research is crucial to delve deeper into the underlying mechanisms by which frailty contributes to adverse outcomes in naSAH. Understanding these pathways could pave the way for the development of specific interventions aimed at counteracting the negative impact of frailty. Such interventions could be tailored to enhance resilience in frail patients, potentially reducing the risk of complications like DCI or hydrocephalus and improving overall recovery and long-term outcomes.

### Limitations

Several limitations of our study should be acknowledged. The retrospective nature of the analysis may introduce selection bias. While the mFI is a validated tool for assessing frailty, it may not capture all aspects of frailty relevant to naSAH patients. Future studies should consider using more comprehensive frailty assessment tools or combining the mFI with other prognostic indicators to provide a more detailed risk profile. We acknowledge that the number of unfavorable outcomes (*n* = 42) in our cohort limits the robustness of the multivariable regression analysis. Including six variables in the model approaches the ‘ten-events-per-variable’ rule, increasing the risk of model overfitting and wide confidence intervals. This limitation should be considered when interpreting the strength and independence of the reported associations. Although the mFI and mRS are conceptually distinct, both include elements of functional status. This overlap introduces a potential for partial redundancy between input and outcome variables. However, as discussed above, most frail patients in our cohort had a premorbid mRS < 3, suggesting that the mFI captured broader aspects of vulnerability beyond functional dependence alone. Nonetheless, some degree of residual confounding cannot be entirely excluded.

## Conclusions

This study demonstrates that frailty, as assessed by the mFI, is associated with a higher risk of poor functional outcome at 6 months following naSAH. Patients with an mFI ≥ 2 experienced a markedly higher rate of unfavorable outcomes. Early frailty assessment may therefore support individualized management—either by identifying patients who may benefit from closer monitoring and supportive care, or by informing decisions to avoid overly aggressive interventions in vulnerable individuals.

## Data Availability

Restrictions apply to the availability of these data due to privacy restrictions.
